# ΔNp63 drives epithelial differentiation in glioma

**DOI:** 10.1002/ctm2.165

**Published:** 2020-08-30

**Authors:** Jia‐Hao Lu, Shuo Li, Wen‐Jie Zhang, Qi Zhang, Chun‐Ming Liu, Shu‐Yang Jiang, Qiao‐Hua Xiong, Xiang‐Yu Meng, Fu‐Bing Wang

**Affiliations:** ^1^ Department of Laboratory Medicine Zhongnan Hospital of Wuhan University Wuhan China; ^2^ D.S.C. Scientific Wuhan China; ^3^ State Key Laboratory of Biocatalysis and Enzyme Engineering School of Life Science Hubei University Wuhan China; ^4^ Department of Urology Zhongnan Hospital of Wuhan University Wuhan China; ^5^ Institut Curie CNRS UMR144 Molecular Oncology Team Paris France

Dear Editor,

Glioma is known for its morphologic and molecular diversity. Clinically documented glioma with epithelial differentiation is rare and linked to poor prognosis with unknown mechanisms.^1^, ^2^ We identified an epigenetically modulated and stress‐related glioma epithelial transcriptional program showing correlation with aggressiveness, recurrence and prognosis in glioma patients, and highlighted ΔNp63 as a likely driver of this cellular plasticity. The study flow diagram is shown in Figure S1.

We applied the GENIE3 algorithm^3^ to transcriptomes of 212 heterogeneous Gene Expression Omnibus brain samples with genes prefiltered by correlation (Pearson's *R* > 0.8) for regulatory network reconstruction. Regulons, namely regulatory relationships, were established concerning 37 core transcription factors (TFs) (top 1% most confident regulations and ≥10 targets). Based on connections among TFs (Table S1), we aggregated the regulons to eight clusters denoted C1 to C8. Nearly all these regulon clusters were univariately associated with prognosis in TCGA glioma patients (Figure S2A). However, only one regulon cluster, the C6 containing 8 TFs and 76 targets, showed independent prognostic significance after adjustment for established glioma prognosticators (Figure S2B). Markers as well as regulators for epithelial differentiation were enriched in C6 genes. We further deconvoluted C6 into two major subsets of genes (Figure S3), with the larger (n = 37) as C6a, which mainly consisted of end targets including epithelial/keratinocyte markers (e.g., cytokeratins), and the smaller (n = 18) as C6b, which mainly consisted of upstream regulators including *TP63* (key regulator of basal/squamous subtype carcinoma) and *MACC1* (coactivator of the receptor tyrosine kinase gene *MET* also involved in basal/squamous subtype carcinoma)^4^ (Table S2). Higher C6b expression was found in glioblastoma (GBM) versus low grade glioma and in recurrent versus primary tumors (Figure S2C). High C6b was an independent predictor for unfavorable prognosis in glioma (Figure S2D).

We developed a network‐based approach to prioritize key C6 regulators using scRNA‐seq data (n = 658). *MET* and its canonical ligand *HGF*, as well as a classical regulator for glioma stemness and metastasis, *CD44*, were considered in addition to C6b genes.^5^ Single cells were clustered to three subpopulations (Figure 1A, left). The highest C6b expression and rate of cells expressing *CD44*, *TP63*, *MET*, and *MACC1* were found in one single‐cell cluster (Figure 1A, middle and right; Table S3). Coexpression dependency network analysis demonstrated *TP63*, *MET*, and *MACC1* as the top three regulators (Figure 1B, left; Table S4). Correlation dynamics analysis revealed higher connectivity among these three genes in samples presenting higher expression, suggesting positive regulatory circuit (Figure 1B, right).

**FIGURE 1:**
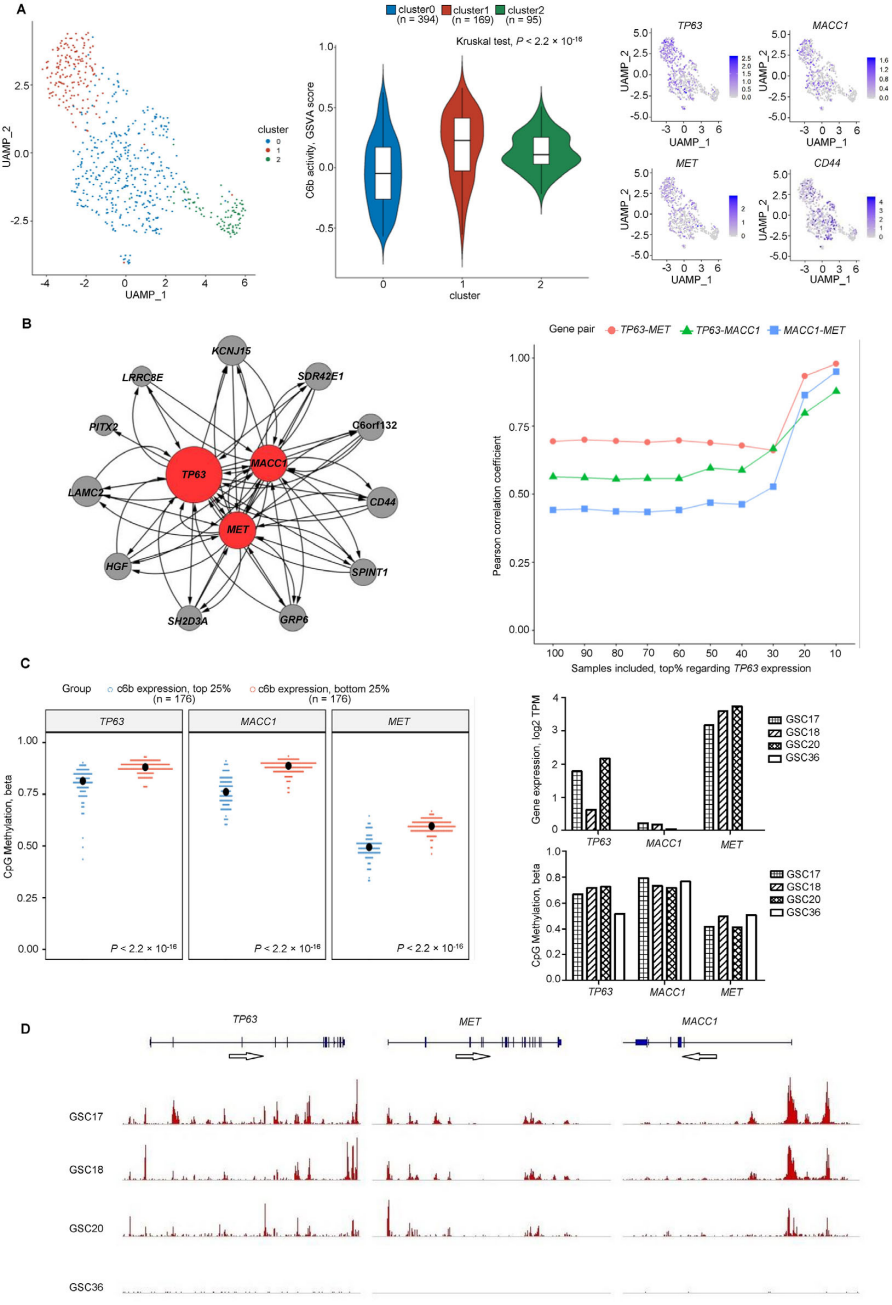
Identification and validation of crucial regulators in C6b. **A,** The single cells could be classified to three clusters. The highest C6b expression level, as well as the highest rate of cells expressing *CD44*, *TP63*, *MET*, and *MACC1*, was found in one single cell cluster. **B,** The regulation dependency network of C6b (Supporting Information Methods), the red cycles represented the top three regulators in the overall regulatory program, and all other genes in gray cycles (**left**). The connectivity dynamics among these three genes in a series of subset of 98 sample of GSC, GBM, and NSC by each time removing the bottom 10% samples in terms of *TP63* expression (**right**). **C,** DNA CpG methylation status of *TP63*, *MET*, and *MACC1* in TCGA glioma samples within the top and bottom 25% in terms of C6b overall expression (GSVA score) (**left**). The expression and DNA CpG methylation status in samples (GSC17, GSC18, and GSC20) with highest global expression of *TP63*, *MET*, and *MACC1*, and a sample (GSC36) expressing none of these genes (**right**). **D,** The CHIP‐seq‐derived H3K27ac histone modification peaks in the above‐mentioned samples

Genetic alterations of *TP63*, *MET*, and *MACC1* were rare in glioma (Figure S4). Thus, an epigenetically mediated activation was suspected. We examined the CpG methylation of *TP63*, *MET*, and *MACC1* in TCGA samples from top and bottom quarter regarding C6b expression. We found for all these genes a significantly lower CpG methylation level in the top quarter (Figure 1C, left). However, in GSC models, there was no difference in CpG methylation between the three models showing highest overall expression (GSC17, GSC18, and GSC20) and the one expressing none of the three genes (GSC36), suggesting a role of other epigenetic mechanisms (Figure 1C, right). We found multiple H3K27ac modification peaks detected around the *TP63*, *MET*, and *MACC1* genomic regions in GSC17, GSC18, and GSC20, but not in GSC36 (Figure 1D). These observations suggest coordinated epigenetics for switching on the expression of the key regulators.

Differential expression and functional impact of p63 isoforms have been reported.^6^ We found the ΔNp63 was the primary isoform expressed in glioma bulk tumors (ΔNp63, n = 589; TAp63, n = 46; Figure S5). Significant positive correlation was found between expression of epithelial markers and ΔNp63 but not TAp63 (Figure 2A), and higher expression of these markers were presented in tumors expressing ΔNp63 compared to those expressing TAp63 at matched level (Figure 2B). No co‐occurrence of these two isoform types was revealed at single cell level, and cells expressing ΔNp63 showed significantly decreased expression and altered exon usage of genes involved in cell cycle and RNA splicing compared to cells expressing TAp63, indicating a stressed phenotype as according to a previous report that stress responses induced by ɣ‐irradiation, hypoxia, and chemotherapy are all associated with downregulation and splicing alteration of genes involved in cell cycling as well as pre‐mRNA splicing machinery in cancer cells (Figure 2C‐E).^7^ We also found in bulk tumors a significant positive correlation between expression of C6 genes involved in response to stress and ΔNp63 but not TAp63 (Figure S6). Thus, selection pressure by hypoxia and chemoradiation may via stress response cascade lead to resistance linked with lineage plasticity such as epithelial differentiation. In glioma cell lines with adequate *TP63* expression (n = 10), we observed a positive correlation between expression of epithelial markers and ΔNp63 but not TAp63 transcripts, coherent with findings in bulk tumors (Figure S7). CRISPR/Cas9‐mediated functional screening revealed that only the two cell lines presenting high *TP63* expression with ΔNp63 as dominant showed significant decrease in cell viability following *TP63* knockout, while almost no effect in others (Figure 2F; Table S5). These data altogether demonstrated stress‐related ΔNp63's isoform‐specific role as a master regulator driving epithelial differentiation in glioma.

**FIGURE 2:**
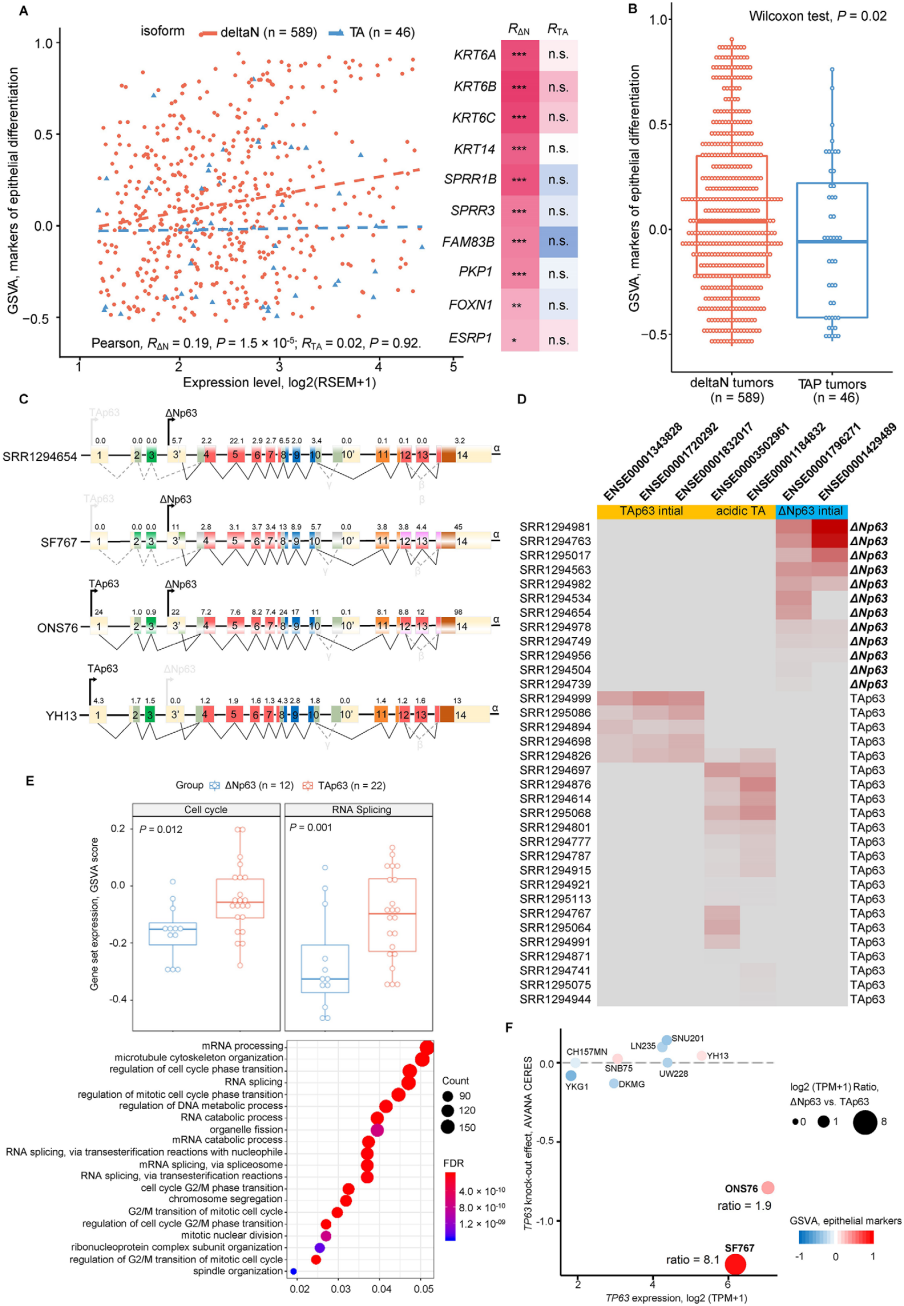
Determination of p63 isoforms and functional implications. **A,** Isoform‐specific correlation between expression of epithelial differentiation markers and ΔNp63/TAp63. Significance: ***P < .001; **P < .01; *P < .05; n.s., nonsignificant. **B,** Differential expression of epithelial markers in tumors expressing ΔNp63 and TAp63. **C,** Representative illustration of p63 isoforms in single GBM cells and glioma cell lines. **D,** Heatmap showing the first seven exons involved in alternative promoter usage in 37 single GBM cells by which the TAp63/ΔNp63 constitution could be determined. **E,** Comparison of the overall expression level (GSVA scores) of genes involved in cell cycle (Gene ontology, GO:0007049) and RNA splicing (GO:0006397, GO:0000377, GO:008380, GO:0000398, and GO:0000375) between the cells presenting TAp63 and ΔNp63 isoforms (**top**). Functional enrichment analysis for Gene Ontology Biological Process (GOBP) concerning genes with significantly different exon usage between the two groups of GBM cells (**bottom**). **F,** Association between *TP63* genetic dependency (CRISPR/CAS9‐mediated knockout effect on cell viability), *TP63* expression, and *TP63* isoform constitution in glioma cell lines with adequate *TP63* expression (log2(TPM+1) > 1.5; n = 10). The two cell lines presenting high *TP63* expression with ΔNp63 as dominant showed significant decrease in cell viability following *TP63* knockout, while almost no effect in others

Tumor heterogeneity and phenotypic plasticity are important aspects of cancer hall marks.^8^ Glioma is well known for its morphologic and molecular diversity.^9^ Our findings suggest an epithelial transcriptional program that exists in glioma and show correlation with disease progression, recurrence, and patients’ survival. Our data highlighted the ΔN isoforms of *TP63* as a likely master regulator driving this cellular plasticity. Potential roles of epigenetic remodeling and cell stress response was also suggested, consistent with similar findings in previous studies.^10^ Glioma cells with epithelial trans‐differentiation potential may contribute to the repertoire of tumor progression, treatment resistance, and metastasis, under natural or therapeutic selection pressure. The novel insights brought by this study form a new perspective and basis for further mechanistic and translational studies aiming to improve the understanding and management of glioma.

One limitation of this work is the retrospective nature of data used. Our multilevel multi‐omics strategy and rigorous statistical design could mitigate its potential effects. Besides, mechanistic exploration is limited at current stage. Nevertheless, the preliminary functional observations reported open a new path to fundamental glioma research.

In summary, we for the first time reported an epigenetically modulated and stress‐related glioma epithelial transcriptional program showing clinical significance, with ΔNp63 as a likely driving master regulator that interacts with *MET* signaling. Our findings deepen the understanding of glioma plasticity and provide novel insights for its management and research.

## CONFLICT OF INTEREST

The authors declare that they have no conflict of interest.

## AUTHOR CONTRIBUTIONS

Jia‐Hao Lu, Shuo Li, Wen‐Jie Zhang, and Qi Zhang were involved in study concept and design, acquisition of data, analysis and interpretation of data, and drafting of the manuscript. Chun‐Ming Liu, Shu‐Yang Jiang, and Qiao‐Hua Xiong were involved in critical revision of the manuscript for important intellectual content and material support. Xiang‐Yu Meng and Fu‐Bing Wang were involved in study concept and design, drafting of the manuscript, critical revision of the manuscript for important intellectual content, statistical analysis, obtaining funding, material support, and study supervision. All authors read and approved the final manuscript.

## FUNDING INFORMATION

Improvement Project for Theranostic Ability on Difficulty Miscellaneous Disease (Tumor) of Zhongnan Hospital of Wuhan University; Grant Number: ZLYNXM202008; National Natural Science Foundation of China; Grant Number: 81672114; Medical Talented Youth Development Project in Health Commission of Hubei Province.

## Supporting information

Supporting Information

## Data Availability

All the data obtained and/or analyzed associated with the current study were available from the corresponding authors upon reasonable request.
