# Supplementary material for: ΔNp63 drives epithelial differentiation in glioma
Source: Clin Transl Med. 2020 Aug 30;10(4):e165. doi: 10.1002/ctm2.165 (PMC7456564; doi:10.1002/ctm2.165)
Supplement: Supplementary file 1 — Supporting Information [file CTM2-10-e165-s001.pdf]

SUPPLEMENTARY MATERIALS AND METHODS

Data collection and preprocessing

Three microarray based transcriptome datasets involving various types of brain tumors, under the accession numbers of GSE44971 (n = 58), GSE50161 (n = 130) and GSE68015 (n = 112), were collected from the NCBI Gene Expression Omnibus (GEO, https://www.ncbi.nlm.nih.gov/geo/) database. Sample annotation files were also collected from the same source. The raw Affymetrix Human Genome U133 plus2 CEL files were downloaded and preprocessed using the robust multi-array average (RMA) algorithm with the oligo Bioconductor package [1]. Principal component analysis (PCA) based removal of outlier and duplicated samples were performed in addition to routine quality control (QC) procedures. Correction of batch effects was performed using the removeBatchEffect function of the limma Bioconductor package [2].

The merged TCGA lower grade glioma (LGG) and glioblastoma (GBM) preprocessed RNA-seq transcriptome at both transcript- and gene- levels (log2 RNA-Seq by Expectation-Maximization, RSEM read counts, n = 702) and Illumina HumanMethylation 450 BeadChip based CpG methylome (beta value, n = 685) datasets, along with *IDH1*, *IDH2*, and *TP53* mutation status (part of the whole exom sequencing derived somatic variant calls, n = 825) and patients' clinical information (n = 1,131), were downloaded from the UCSC Xena server (https://xena.ucsc.edu/).

Raw full length RNA sequencing data (SMART-Seq) of 677 flow cytometry sorted single tumor cells from 5 GBM patients were downloaded from the NCBI Short Read Archive (SRA) database, under the study accession number SRP042161. The raw files downloaded were first reverted to standard paired FASTQ files using the fastq-dump function of the SRA toolkit, and then aligned to hg38 human reference genome using the STAR package [3]. Gene and exon level raw read counts were obtained using the featureCounts function of the Subread package [4].

The preprocessed RNA-seq transcriptome (fragments per kilo base per million mapped reads, FPKM normalized read counts), Illumina HumanMethylation 850 BeadChip based CpG methylome (beta value), and H3K27 acetylation ChIP-seq (AQUAS pipeline processed, with MACS2 peak calling following BWA alignment and QC)

datasets of 44 glioblastoma stem cell (GSCs) models, 50 primary GBM samples, and 10 neural stem cells (NSCs) were download from GEO database under the accession numbers of GSE119834, GSE119774, and GSE119755, respectively [5].

The preprocessed gene-level (log2 TPM normalized) and transcript-level (RSEM normalized) RNA-seq transcriptoms, as well as functional dependency scores revealed by CRISPR/Cas9 mediated *TP63* knock-out screening (version 20Q2) of 10 brain cancer cell lines with adequate *TP63* expression (log2 TPM > 1.5; YKG1, CH157MN, DKMG, SNB75, LN235, UW228, SNU201, YH13, ONS76, and SF767) were downloaded from the Cancer Dependency Map data portal (https://depmap.org/portal/).

Computational reconstruction of gene regulatory programs

A merged microarray-based transcriptome dataset containing 212 heterogeneous normal brain (n = 22, collected from multiple brain regions) and brain tumor samples (adamantinomatous craniopharyngioma, n = 15; choroid plexus papilloma, n = 5; ependymoma, n = 46; GBM, n = 34; medulloblastoma, n = 22; pilocytic astrocytomas, n = 64; primitive neuroectodermal tumor, n = 4) was used for the computational reconstruction of gene regulatory programs reserved in normal brain and/or brain tumor samples. After the above mentioned preprocessing, we removed less varying probes (the bottom 20% sample variance) and mapped the probes to genes. For genes with multiple corresponding probes, only the value of the most varying probe was retained. A Pearson correlation matrix was then built using this gene level transcriptome, and only genes pairs with adequate co-expression connectivity (Pearson correlation coefficient > 0.8) were retained. Then, genes with fewer co-expression partners (number of partners < 50) were further removed. The genes passed all the above filtering criteria were included for gene regulatory program reconstruction using the Bioconductor GENIE3 packages. Only the top 1% estimated regulation relationships and TFs with ≥ 10 targets (n = 37) were consisted as the final regulatory program, i.e. the core regulons in brain tissue and tumors. Finally, taking the regulatory connections among TFs, we identified eight inter-correlated regulon clusters as a higher level representation of regulatory relationships identified (Supplementary Table 1). These regulon clusters were denoted as C1 to C8.

Analysis of regulons' prognostic significance in glioma patients

The merged TCGA LGG & GBM dataset (LGG, n = 530; GBM, n = 172) was used for investigating the regulons' prognostic significance in glioma. The overall survival (OS) was the outcome of interest.

The activity of each regulon cluster was calculated using the gene set variation analysis (GSVA) algorithm implemented with the GSVA Bioconductor package [6]. In brief, for a given regulon cluster, its regulation activity in each sample was calculated as the positive targets' sample-specific enrichment score subtracting that of the negative targets. The patients were divided to two groups by each regulon cluster's activity and these grouping factors were subjected to univariate and multivariate Cox proportional hazard model analyses. The covariates for adjustment in multivariate model included the established prognostic factors in glioma patients, namely the disease stage as LGG vs. GBM, the *IDH1*, *IDH2*, and *TP53* mutation status, and the molecular subtypes. GSVA based enrichment scores of specific markers for the 3 major molecular subtypes (pro-neuronal, mesenchymal, and classical) were calculated for each patient as a proxy of similarity to these molecular subtypes [7].

Regarding the regulon cluster 6, i.e. C6, the regulon cluster showed independent prognostic significance uniquely among all regulon clusters, we further deconvoluted it into two highly intra-correlated subsets (modules) and removed all other genes unrelated to these two cores, using the ConsensusClusterPlus Bioconductor package (Supplementary Figure 2) [8]. We defined the larger subset (n = 37) as C6a, which mainly consisted of end targets including markers associated with epithelial/squamous differentiation (*KRT4*, *KRT5*, *KRT6A*, *KRT6B*, *ITGB6*, *LAMB3*, *COL17A1*, etc), and the smaller set (n = 18) as C6b which mainly consisted of upstream regulators including *TP63*, a key regulator of basal/squamous subtype epithelial carcinoma, as well as *MACC1*, a transcriptional activator of *MET* that is also involved in basal/squamous subtype carcinoma [9]. The prognostic significance of these two subsets was further tested in multivariate Cox regression model as described above. The C6b activity was compared against tumor stage (LGG vs. GBM) and tumor recurrence (primary vs. recurrent) using Wilcoxon test.

Network-based prioritization of key regulators

The C6b regulon, whose activity was independently associated with glioma patients' OS as well as tumor stage and tumor recurrence, represents the focus of our subsequent investigation.

The gene level raw read count table of 677 single tumor cells from 5 glioblastoma patients along with annotation files were loaded into R and subjected to the routine preprocessing and integration workflow of the Seurat v3.1.2 Bioconductor package [10,11]. The intrinsic variation attributed to cell cycle was regressed out [12].

We developed a network-based approach to prioritize the key regulators in C6b regulon, using the preprocessed GBM single cell transcriptome data (n = 658). The single cell data was used because it is free of micro-environment contamination and large in sample size. *MET* along with its canonical ligand *HGF*, as well as a classical regulator for glioma stemness and metastasis, *CD44*, was also considered in addition to the C6b regulon genes. We first identified all the significantly co-expressed gene pairs (false discovery rate, FDR < 0.05) using Cohen's kappa agreement analysis, following dichotomization by expressed or not. We then tested the dependency of each co-expression gene pair on a third gene's expression status (expressed or not) using Fisher's exact test. In brief, if the proportion of cells showing expression of both genes was significantly higher in cells expressing the third gene than in cells not expressing the third gene (FDR < 0.05), a regulatory dependency of the gene pair on the third gene was created. This dependency underscores the third gene's role in regulating the co-expression of the gene pair, either by regulating directly both the two genes or by mediating the regulatory effects between the two genes. In a network setting, two directed edges would be created from the two genes of the co-expression pair to the third gene, and the odds ratio (OR) of cells showing expression of both genes would be considered as the edge weight. The edge weights were then min-max normalized. The genes were finally ranked according to the their weighted in-degrees. The larger weighted in-degree suggests larger importance in the overall regulatory program.

For the top three most important regulators according to the regulation dependency network, ie the *TP63*, *MET*, and *MACC1*, we further examined the connectivity

dynamics among these three genes. We created a series of subsets of 98 samples of GSC, GBM, and NSC by each time removing the bottom 10% (fixed number, n = 10) in terms of *TP63* expression, and calculated the Pearson correlation coefficients among these three genes for each subset. Larger correlation coefficients in samples with higher *TP63* expression would suggest the existence of regulatory circuit among these regulators.

Dissecting the epigenetic landscape of *TP63*, *MET*, and *MACC1* in glioma

Genetic alterations of *TP63*, *MET* and *MACC1* were rare in glioma patients (Supplementary Figure 3). An epigenetic activation was suspected. We examined the DNA CpG methylation status of *TP63*, *MET* and *MACC1* in TCGA glioma samples within the top and bottom 25% in terms of C6b overall expression (GSVA score). We also examined the CHIP-seq derived H3K27AC histone modification peaks, a marker of active enhancer elements, in the proximity of *TP63*, *MET* and *MACC1* in three GSC samples (GSC17, GSC20, and GSC18) where they were highly expressed and one GSC sample (GSC36) where none of the them were expressed, as well as the DNA CpG methylation status (data from GSE119834, GSE119774, and GSE119755).

Determination of p63 isoforms and functional implications

Considering different p63 isoforms could have distinct functional impacts to cell behavior, we characterized the p63 isoform distribution in bulk GBM samples, single GBM cells, and brain cancer cell lines (data collection and preprocessing described above). The p63 protein has two major subgroups, the TAp63 and the ΔNp63. The difference between the two is the inclusion of an acidic transcription activation (TA) domain in the N-terminus in TAp63 isoforms but not in ΔNp63 isoforms. The *TP63* exon annotation was retrieved from the neXtProt knowledge database [13]. The presence and quantification of TAp63 and ΔNp63 isoforms were determined by transcript-level transcriptome for bulk TCGA data and by the expression of first seven exons (by chromosome location, namely, the TAp63-specific initial exons, ENSE00001343828, ENSE00001720292, and ENSE00001832017; the N-terminus TA exons, ENSE00003502961 and ENSE00001184832; and the ΔNp63-specific initial exons, ENSE00001796271 and ENSE00001429489) for the single cell data. At bulk

level, we measured the isoform-specific correlation between expression of epithelial markers (squamous cytokeratin's, epithelial-specific TF *FOXN1*, epithelial-specific splicing factor *ESRP1*, etc) and ΔNp63 as well as TAp63. At single cell level, at least one of these seven exons were covered in 37 GBM single cells where the TAp63/ΔNp63 constitution could be determined. We extracted the expression of all the *TP63* exons (TPM) for these cells and determined the major p63 isoform in each cell. We compared the overall expression level of genes involved in cell cycle (Gene ontology, GO:0007049) and RNA splicing (GO:0006397, GO:0000377, GO:008380, GO:0000398, and GO:0000375), via GSVA scores, between the cells presenting TAp63 and ΔNp63 isoforms. We performed hypergeometric test based functional enrichment analysis for Gene Ontology Biological Process (GOBP) concerning genes with significantly different exon usage between the two groups of GBM cells (at least one exon's expression level was significantly different between the two groups by Wilcoxon test, $P < 0.05$ as significance level). Correlation between expression of C6 genes involved in response to stress (*GPX2*, *SH3RF2*, and *GPR87*) and ΔNp63 as well as TAp63 was also investigated in bulk TCGA tumors.

We validated the isoform-specific correlation between expression of epithelial markers and ΔNp63 or TAp63 isoforms in 10 glioma cell lines with adequate *TP63* expression (log2 TPM > 1.5) as mentioned above. We further analyzed the association between *TP63* expression, *TP63* isoform constitution, and genetic dependency on *TP63* (functional impact following CRISPR/Cas9 mediated *TP63* knockout) in these cell lines.

REFERENCES

1. Irizarry RA, Hobbs B, Collin F, et al. Exploration, normalization, and summaries of high density oligonucleotide array probe level data. *Biostatistics*. 2003;4(2):249–264. doi:10.1093/biostatistics/4.2.249

2. Smyth G. Limma: linear models for microarray data. In: Gentleman R., Carey V., Dudoit S., Irizarry R., Huber W., editors. Bioinformatics and Computational Biology Solutions Using R and Bioconductor. New York: Springer; 2005. pp. 397–420.

3. Dobin A, Davis CA, Schlesinger F, et al. STAR: ultrafast universal RNA-seq aligner. *Bioinformatics*. 2013;29(1):15–21. doi:10.1093/bioinformatics/bts635

4. Liao Y, Smyth GK, Shi W. featureCounts: an efficient general purpose program for assigning sequence reads to genomic features. *Bioinformatics*. 2014;30(7):923–930. doi:10.1093/bioinformatics/btt656

5. Mack SC, Singh I, Wang X, et al. Chromatin landscapes reveal developmentally encoded transcriptional states that define human glioblastoma. *J Exp Med*. 2019;216(5):1071–1090. doi:10.1084/jem.20190196

6. Hänzelmann S, Castelo R, Guinney J. GSVA: gene set variation analysis for microarray and RNA-seq data. *BMC Bioinformatics*. 2013;14:7. Published 2013 Jan 16. doi:10.1186/1471-2105-14-7

7. Teo WY, Sekar K, Seshachalam P, et al. Relevance of a TCGA-derived Glioblastoma Subtype Gene-Classifier among Patient Populations. *Sci Rep*. 2019;9(1):7442. Published 2019 May 15. doi:10.1038/s41598-019-43173-y

8. Wilkerson MD, Hayes DN. ConsensusClusterPlus: a class discovery tool with confidence assessments and item tracking. *Bioinformatics*. 2010;26(12):1572–1573. doi:10.1093/bioinformatics/btq170

9. Stein, U., Walther, W., Arlt, F. *et al.* MACC1, a newly identified key regulator of HGF-MET signaling, predicts colon cancer metastasis. *Nat Med* **15,** 59–67 (2009). https://doi.org/10.1038/nm.1889

10. Stuart T, Butler A, Hoffman P, et al. Comprehensive Integration of Single-Cell Data. *Cell*. 2019;177(7):1888–1902.e21. doi:10.1016/j.cell.2019.05.031

11. Butler A, Hoffman P, Smibert P, Papalexi E, Satija R. Integrating single-cell transcriptomic data across different conditions, technologies, and species. *Nat Biotechnol*. 2018;36(5):411–420. doi:10.1038/nbt.4096

12. Scialdone A, Natarajan KN, Saraiva LR, et al. Computational assignment of cell-cycle stage from single-cell transcriptome data. *Methods*. 2015;85:54–61. doi:10.1016/j.ymeth.2015.06.021

13. Lane L, Argoud-Puy G, Britan A, et al. neXtProt: a knowledge platform for human proteins. *Nucleic Acids Res*. 2012;40(Database issue):D76–D83. doi:10.1093/nar/gkr1179

Figure S1. Study flow chart.

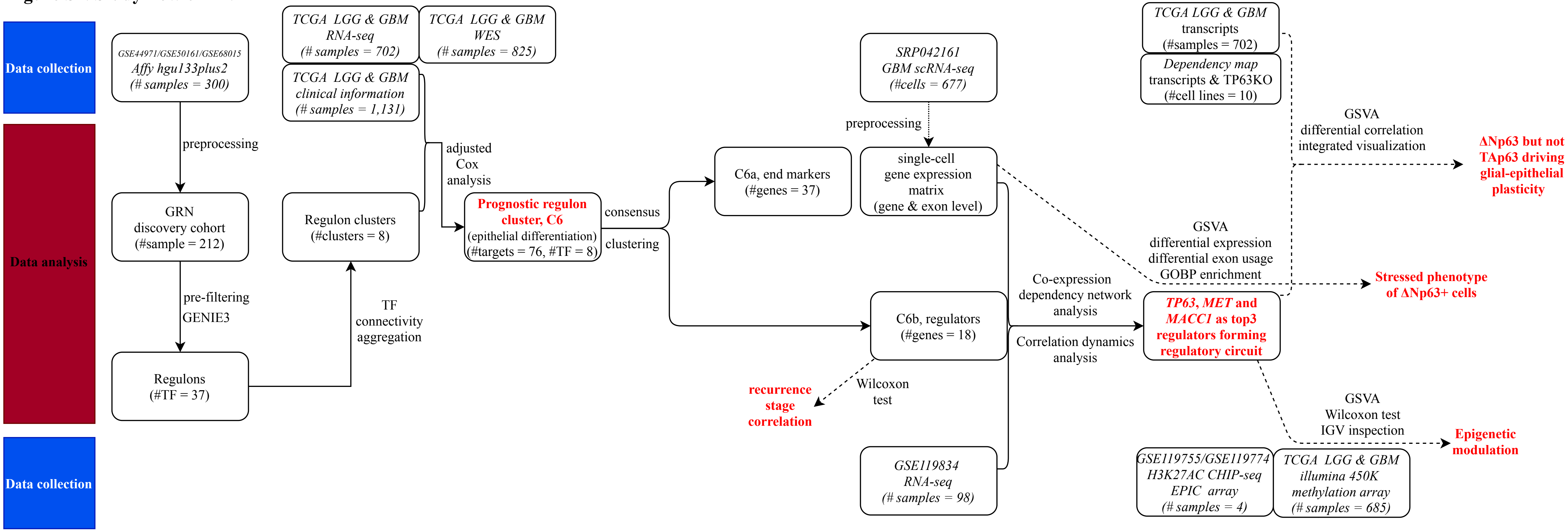

Figure S2. Clinical significance of regulon clusters and C6b module.

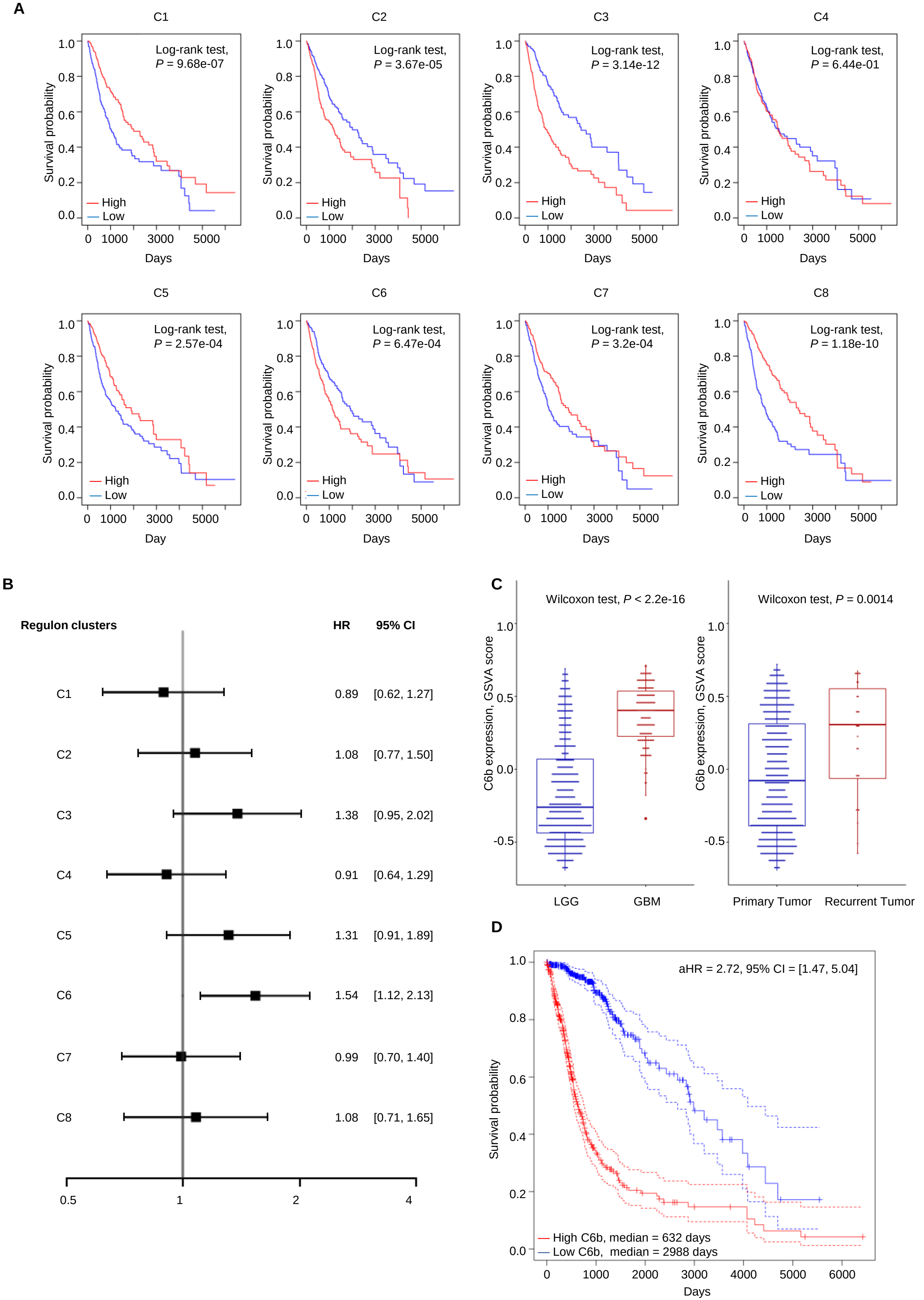

Figure 3. Consensus clustering for C6 genes (k = 10)

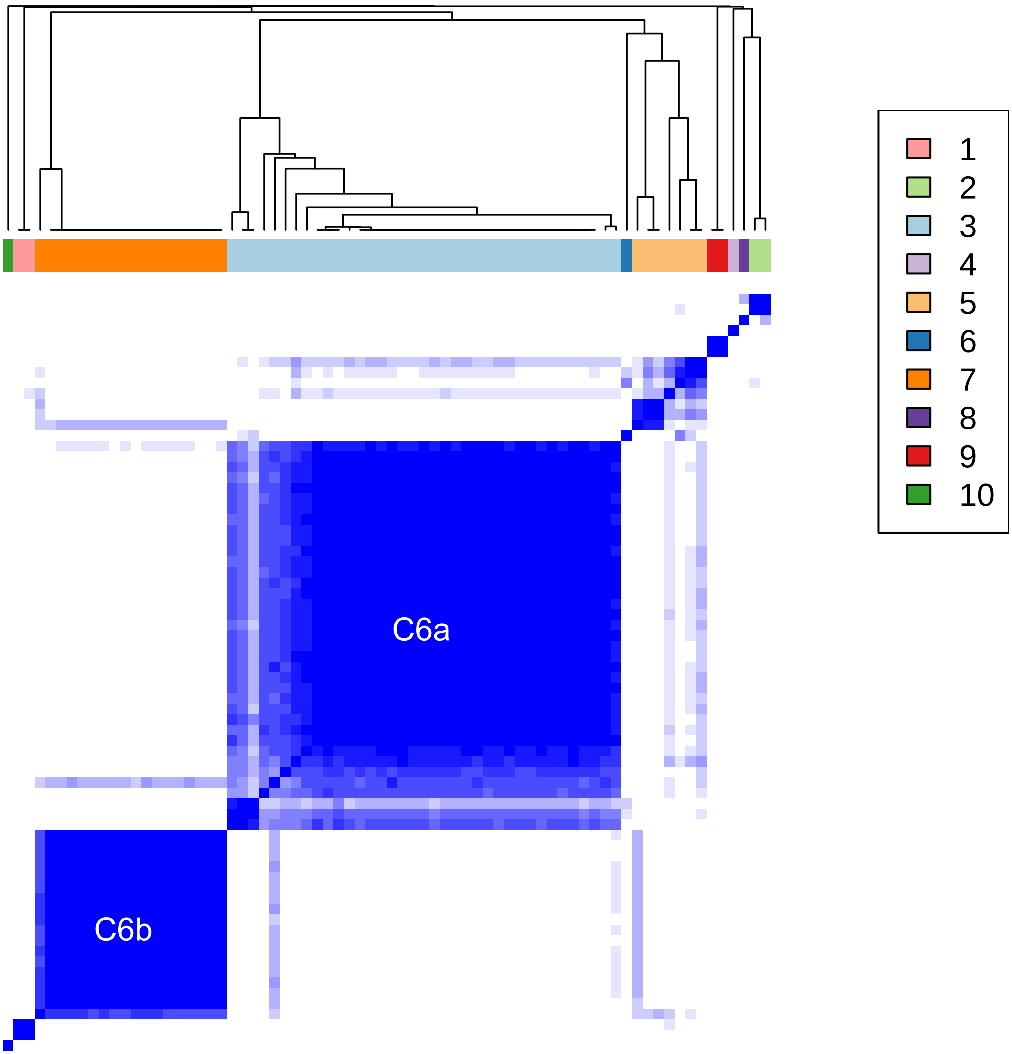

Figure S4. Genetic alterations of *TP63*, *MACC1*, and *MET* in glioma cohorts.

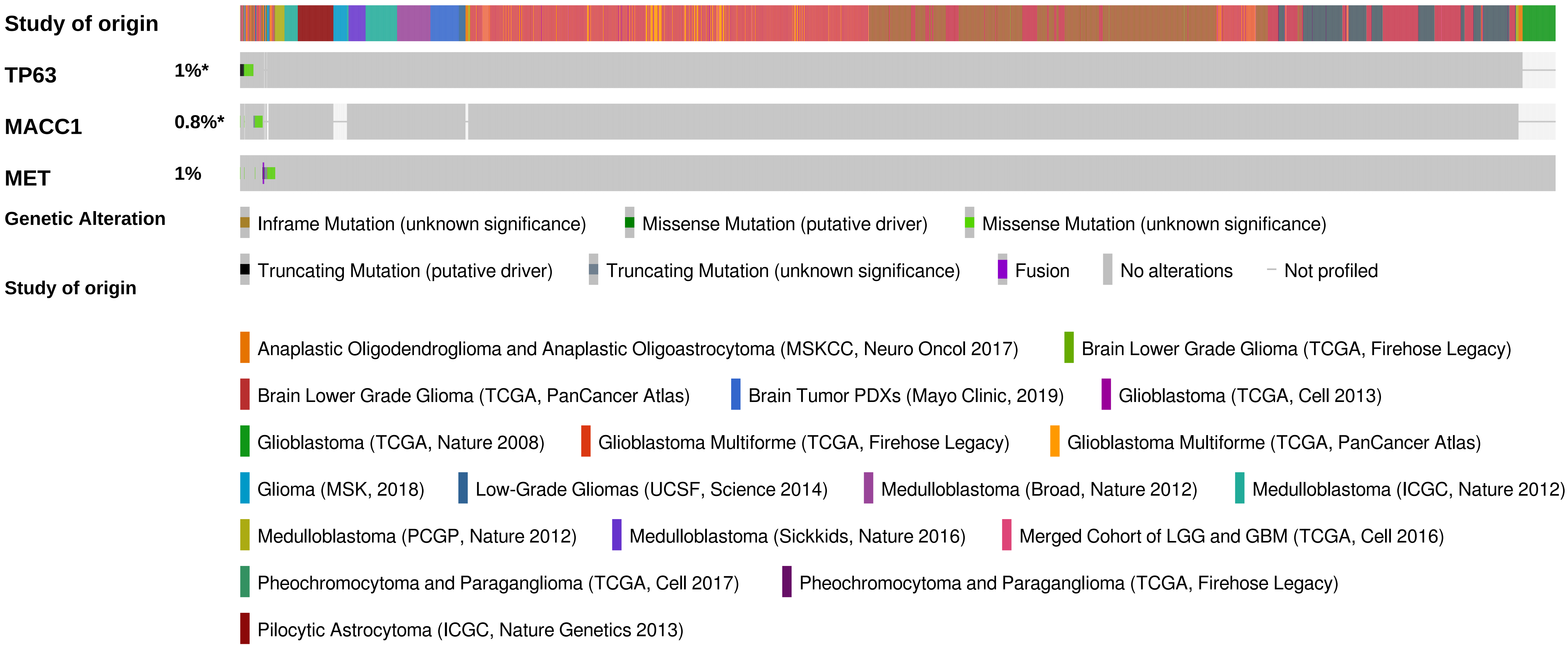

Figure S5. *TP63* isoform distribution in TCGA LGG/GBM dataset.

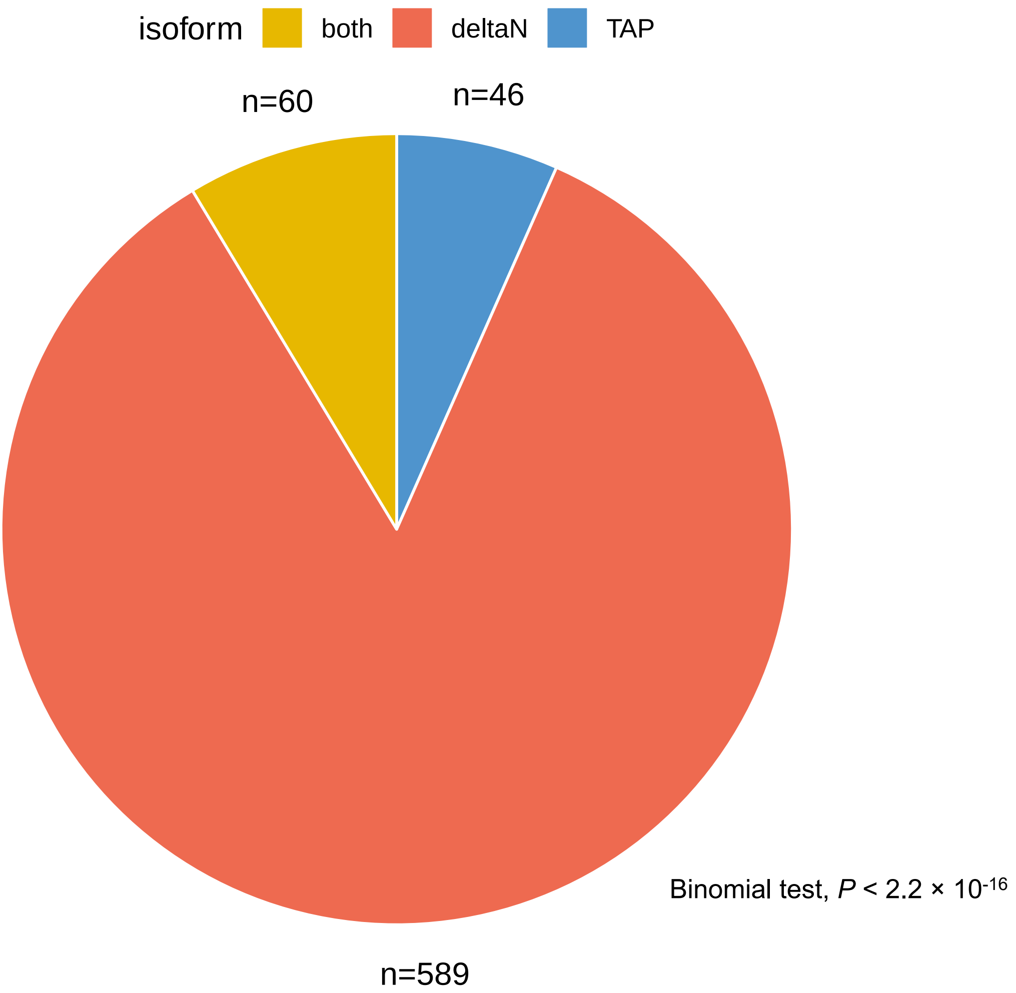

Figure S6. Correlation between expression of stress response markers and p63 isoforms in TCGA LGG/GBM dataset.

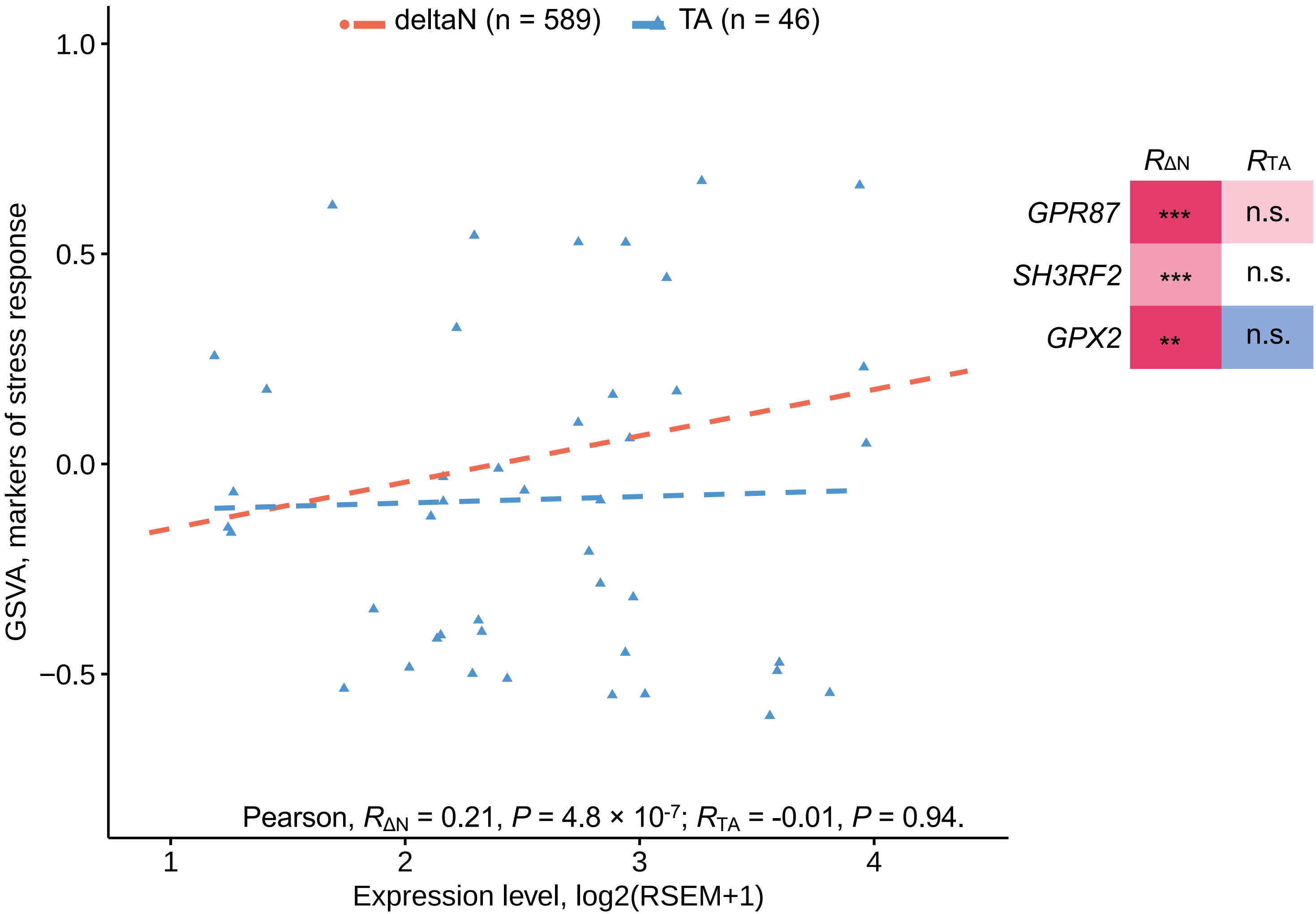

Figure S7. Correlation between expression of epithelial markers and *TP63* transcripts of deltaN or TA isoforms.

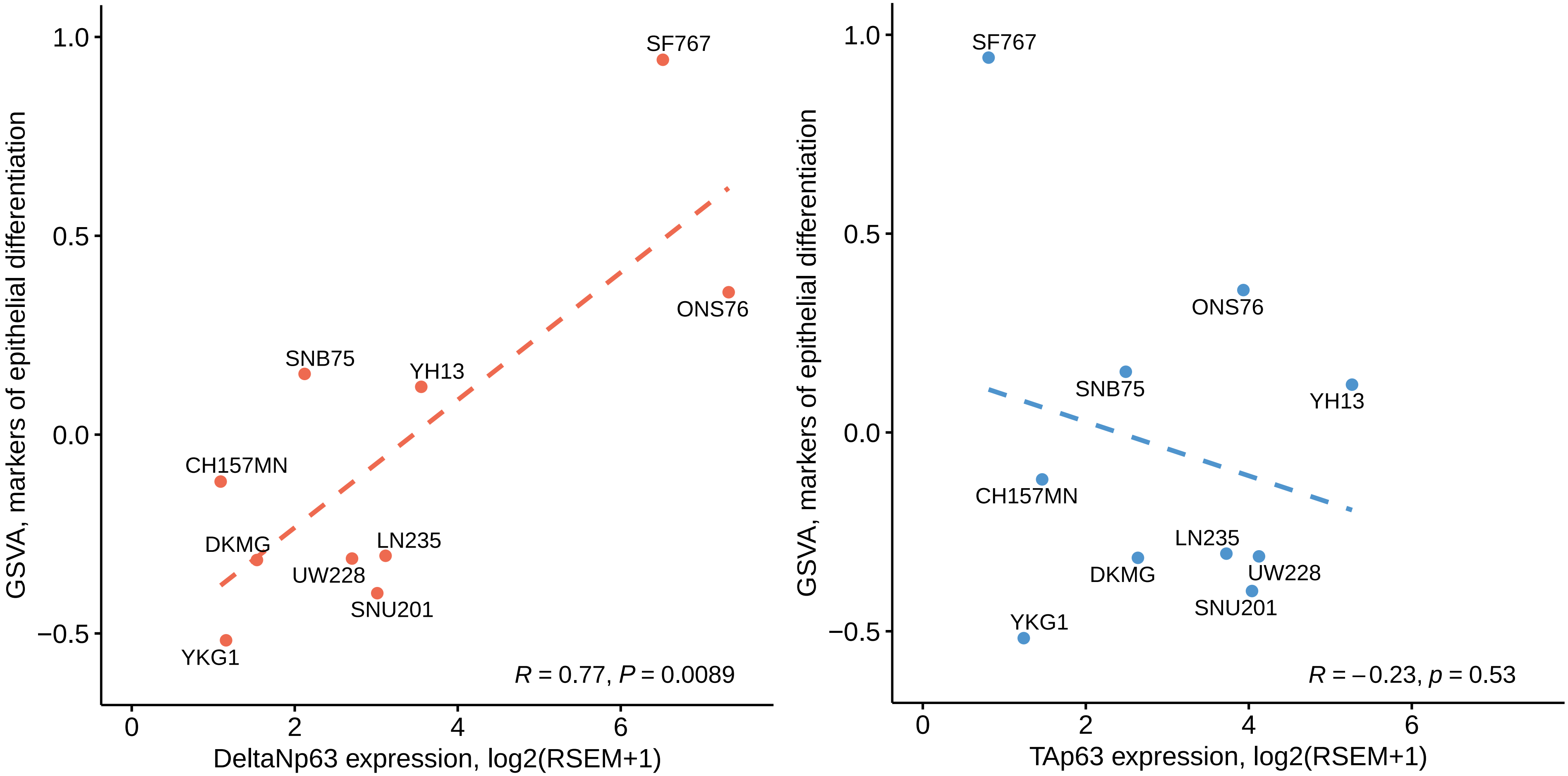

Table S1 : Transcriptional factors in regulon clusters

Cluster_ID	Transcriptional factors included
Cluster1	*ZNF507; MIER2; ZNF664; MIER1*
Cluster2	*MSX1; FOXJ1; RFK2*
Cluster3	*SP100; IKZF1; ELF1; FLI1; TFEC*
Cluster4	*ZNF282; FIZ1; ARID1A; MEF2A*
Cluster5	*DUX4; ZNF771*
Cluster6	*DLX4; TP63; GRHL2; SP6; PITX2; ZBED2; FOXN1; PITX1*
Cluster7	*E2F4; ATF7; ZNF317; HMBOX1; KMT2A*
Cluster8	*ZFP91; ATRX*

Table S2. C6 modules by consensus clustering

Module ID	Genes involved
C6a	*ANXA8; CALML3; CEACAM6; CLCA2; COL17A1; DSG3; ESRP1; FAM83B; FOXN1; FXYD3; GRP87; GPX2; GRHL2; ITGB6; KRT13; KRT14; KRT15; KRT5; KRT6A; KRT6B; KRT6C; LAMB3; LGALS7; LGALS7B; LYPD3; MUC15; PKP1; PKP3; PRSS8; RAB25; S100A14; SCEL; SERPINB5; SH3RF2; SPINK7; SPRR1B; SPRR3*
C6b	*C6orf132; CGB7; GBP6; KCNJ15; KRT23; KRT80; LAMC2; LRRC8E; MACC1; NTF4; PITX1; PITX2; SDR42E1; SH2D3A; SP6; SPINT1; TP63; ZBED2*

Table S3. Proportion of cells expressing selected gene (combinations) in three single cell clusters

Genes expressed	Cluster0	Cluster1	Cluster2
TP63	0.71	0.96	0.37
CD44	0.66	0.81	0.37
HGF	0.43	0.72	0.07
MET	0.66	0.84	0.19
TP63 & CD44	0.51	0.77	0.09
TP63 & HGF	0.36	0.67	0.03
TP63 & MET	0.54	0.80	0.07
CD44 & HGF	0.32	0.61	0.01
CD44 & MET	0.49	0.66	0.06
HGF & MET	0.34	0.58	0.01
TP63 & CD44 & HGF	0.27	0.57	0.01
TP63 & CD44 & MET	0.41	0.63	0.03
TP63 & HGF & MET	0.30	0.55	0.00
CD44 & HGF & MET	0.27	0.49	0.00
TP63 & CD44 & HGF & MET	0.23	0.47	0.00

Table S4: Regulatory strength (weight) for nodes in C6b derived co-expression dependency network.

Regulators	Weight
TP63	12.29
MET	5.74
MACC1	5.67
CD44	3.69
LAMC2	3.47
KCNJ15	3.35
SDR42E1	2.51
HGF	2.32
GBP6	2.31
SPINT1	2.25
C6orf132	2.22
SH2D3A	1.70
LRRC8E	0.13
PITX2	0.09

Table S5. *TP63* isoform expression in glioma cell lines

Cell Line	ΔNp63	TAp63	TPM ratio
CH157MN	1.13	1.76	0.64
DKMG	1.9	5.23	0.36
YH13	10.73	37.5	0.29
SF767	90.58	0.75	120.77
SNB75	3.35	4.62	0.73
YKG1	1.23	1.36	0.90
LN235	7.66	12.22	0.63
SNU201	7.07	15.44	0.46
ONS76	159.28	14.28	11.15
UW228	5.51	16.45	0.33
